# Platelet Count and Platelet Indices as Predictive Markers of COVID-19 Mortality

**DOI:** 10.33549/physiolres.935645

**Published:** 2026-02-01

**Authors:** Zuzana KOZELOVÁ, Dominika JARČUŠKOVÁ, Ladislav TOMČO, Laura LACKOVÁ, Zuzana KOZÁROVÁ, Marcela VALIKOVÁ BAVOĽÁROVÁ, Lenka RUŠINOVÁ, Miriam KOZÁROVÁ

**Affiliations:** 1 Fourth Internal Department, Medical Faculty P.J. Safarik University, Košice, L. Pasteur University Hospital, Slovak Republic; 2 First Psychiatric Department Medical Faculty P.J. Safarik University, Košice, Slovak Republic; 3 Department of Epidemiology, Medical Faculty, P.J. Safarik University, Košice, Slovak Republic, Hygiene Service L. Pasteur University Hospital, Košice, Slovak Republic; 4 Medical Faculty P.J. Safarik University, Košice, Slovak Republic; 5 Department of Occupational Medicine and Clinical Toxicology, Medical Faculty, P.J. Safarik University, Košice, Slovak Republic; 6 Department of Clinical Oncology, Stefan Kukura Hospital, Michalovce, Slovak Republic

**Keywords:** Platelets, COVID-19, Mean platelet volume, Neutrophil to lymphocyte ratio, Mortality

## Abstract

The aim of the study was to analyze changes in platelet count and function in hospitalized COVID-19 patients and compare them with non-COVID-19 patients, focusing on the association between platelet indices and mortality beyond the known link between neutrophil-to-lymphocyte ratio (NLR) and COVID-19 severity. The study sample consisted of 572 patients, out of which 472 were hospitalized with COVID-19 infection from 15^th^ October 2021 to 30^th^ April 2022 in Louis Pasteur University Hospital Kosice, Slovak Republic and 100 represented the control group without COVID-19 infection. COVID-19 positive patients (n=472) had significantly larger size of platelets (MPV 9.2±1.4 vs. 8.8±1.2, p=0.002) and therefore a higher percentage of platelets larger than 12 fl (P-LCR 33.7 % vs. 24.8 %, p=0.002) than patients in the control group (non-COVID19). The statistically significant relationship was between mortality in patients with COVID-19 infection (n=122) and the larger size of the platelets (MPV), higher platelet large cell ratio, (P-LCR), higher PLT/MPV ratio, higher platelet distribution width to plateletcrit ratio (PDW/PCT), higher neutrophil-to-lymphocyte ratio (NLR) (p<0.001, respectively) and lower platelet count (PLT) and lower plateletcrit (PCT) (p=0.006; p=0.028; respectively). In multivariable logistic regression analysis, a significant positive correlation between mortality, MPV (OR 2.29; 95 % CI 1.70–3.08, p<0.001) and age (OR 1.06; 1.03–1.08, p<0.001) was observed. When NLR was included into this model, MPV was stronger predictor of mortality (OR 2.48; 95 % CI 1.79–3.43, p<0.001) compared to NLR (OR 1.06; 95 % CI 1.03–1.08, p<0.001) and age (OR 1.04; 95 % CI 1.02–1.07, p<0.001). MPV is a strong and independent predictor of mortality in hospitalized COVID-19 patients, demonstrating superior prognostic value compared to established association between neutrophil-to-lymphocyte ratio (NLR) and COVID-19 severity. As a simple and routinely available parameter from standard blood count, MPV may serve as a practical and accessible tool for early risk stratification in the clinical management of COVID-19.

## Introduction

Even after 5 years since the World Health Organization declared the COVID-19 pandemic caused by the coronavirus SARS-CoV2, there are still numerous unresolved questions across all medical specializations [1]. COVID-19 is considered to be a multiorgan, multivascular disease with a variable clinical course and complications, including thrombotic ones, which are closely related to the presence of risk factors.

The role of platelets (PLT) in the process of hemostasis is clearly described, however, they also play a significant role in the innate immunity and the inflammatory response by being involved in cytokine production and modulating immune cell interaction [2,3]. Increased production of cytokines and acute phase reactants affects megakaryopoiesis, leading to the release of immature platelets from the bone marrow, and this results in alteration of some platelet indices.

Platelet indices include mean platelet volume (MPV, reference range 6.8–10.2 fl), platelet distribution width (PDW, reference range 15.5–17.1 %), plateletcrit (PCT, reference range 0.19–0.36 %), and platelet-large cell ratio (P-LCR) that is used to evaluate the proportion of large platelets in the blood (percentage of PLT with a volume >12 fl, reference range 15–35 %). These readily available and inexpensive parameters of platelets automatically detected by cytoflow analyzers are part of the blood count examination and together with some ratios, e.g. PLT/MPV, PDW/PCT, may be helpful in assessing the platelet production and function.

According to several authors, PLT and plateletcrit (PCT) decrease, MPV increases (to values more than 11fl), PDW increases (to values more than 25 %), and P-LCR increases (more than 35 %) during COVID-19 infection [2–4]. Platelet count determined on the 1^st^ to 3^rd^ day of hospitalization in patients with COVID-19 infection reflects not only platelet consumption, but also viral suppression of the bone marrow, destruction of PLT by the immune system, and possible aggregation of PLT in the lungs [5]. Increased MPV indicates larger platelets that are more reactive and synthesize more cytokines and thromboxane A2. Their granules are denser than those of smaller PLT. MPV is a marker of the severity and prognosis of inflammation and is also significantly increased in sepsis. Some authors indicate that platelet size expressed by the MPV parameter is a better predictor of mortality in the case of COVID-19 than the neutrophil to lymphocyte ratio (NLR) that has been widely reported as a reliable marker of systemic inflammation and has shown a consistent association with disease severity and poor outcomes in patients with COVID-19 [4,6,7,8,9]. *In vitro* mechanistic studies emphasize that the interaction of the SARS-CoV2 virus with megakaryocytes alters the platelet transcriptome [2]. There is the direct and indirect interaction between platelets and SARS-CoV2 virus, the virus contributes to activation of programmed cell death pathway in platelets and consequent extracellular vesicle release [10,11].

The aim of the study is to analyze changes in the platelet count and function, which can influence COVID-19 severity and prognosis of the patients hospitalized at reprofiled COVID-19 workplace in Louis Pasteur University Hospital in Kosice and compare them with a group of patients who did not have COVID-19 disease. In the study sample of patients with COVID-19 infection, we analyzed the association of selected platelet indices with mortality beyond the established association between neutrophil-to-lymphocyte ratio (NLR) and COVID-19 severity.

## Patients and Methods

It was retrospective biomedical research approved by the Ethics Committee of L. Pasteur University Hospital, Rastislavova 43, Košice under number 2022/EK/06055. All study patients were of Caucasian ethnicity with a homogeneous genetic background from the East Slovak population. The study sample included 572 patients, of which 472 were hospitalized in the period from 15^th^ October 2021 to 30^th^ April 2022 at reprofiled COVID-19 workplace in L. Pasteur University Hospital in Košice, Slovak Republic, whereas 100 patients represented the control group without COVID-19 infection. COVID-19 negative patients were hospitalized at L. Pasteur University Hospital in Košice, Slovak Republic (Neurologic Dept.) from 1^st^ Aug to 1^st^ Dec 2022 with vertebral algic problems. The exclusion criteria were any concomitant acute or chronic inflammatory or infectious diseases, malignancy, severe endocrinopathies, and chronic renal insufficiency (eGF <60 ml/min/1.73 m^2^).

Abstracted data included demographics, hospital admission/discharge date, date of death, the primary diagnosis on admission. Venous blood samples were collected at the time of hospital admission. Complete blood counts were performed using an automated hematology analyzer (Sysmex XN-1000, Sysmex Corporation, Kobe, Japan). Laboratory parameters including PLT, PDW, MPV, PCT, differential white blood cell count to count NLR as the absolute neutrophil count to the absolute lymphocyte count ratio, were collected. In-hospital mortality was the primary end point.

All statistical analyses were performed using SigmaStat 13.0 program (Systat Software Inc., USA). Continuous variables were expressed as medians with interquartile ranges (IQR) or means ± standard deviations (SD), as appropriate. Categorical variables were summarized as absolute frequencies and percentages. The Shapiro-Wilk test was used to assess normality of distribution for continuous variables. Associations between the tested variables were examined through subgroup comparisons and correlation analyses. Between-group comparisons (according to COVID-19 status, mortality) were carried out using the Mann-Whitney U test for two-group comparisons. Correlations with continuous variables (age, PLT, MPV, PDW, P-LCR) were assessed using Pearson’s correlation coefficients. To identify independent predictors of mortality, multiple logistic regression models were constructed. The dependent variable was mortality. Independent variables included age, various platelet indices and NLR. All statistical tests were two-sided, and *p*-values <0.05 were considered statistically significant.

## Results

In the group of patients with COVID-19 (n=472, 45 % men), the mean age was 68.7±15.1 years. Women with COVID-19 infection (n=260) had a mean age 70.1±15.1 years, men (n=212) 67.0±5.0 years (p=0.026). Arterial hypertension was present in 65.3 % of patients (n=308), dyslipidemia in 19.7 % (n=93), coronary heart disease in 37.5 % (n=177), previous cerebral stroke in 21.8 % (n=103), and type 2 diabetes mellitus in 26.9 % (n=127). There were 48 men in the control group (48 %) with the mean age of 62.5±13.8 years and 52 women (52 %) with an average age of 66±7.7 (p=0.131).

The number of platelets (PLT) in the whole study sample significantly negatively correlated with the age of the patients (r=−0.210, p=0.036) and with the platelet size expressed by MPV (r=−0.305, p=0.002).

COVID-19 positive patients (n=472) had significantly larger size of platelets (MPV 9.2±1.4 vs. 8.8±1.2, p=0.002) and therefore a higher percentage of platelets larger than 12 fl (P-LCR 33.7 % vs. 24.8 %, p=0.002) than patients in the control group (non-COVID19). No statistically significant association was observed between platelet count, plateletcrit and the rate of anisocytosis of PLT (platelet distribution width, PDW) in relation to COVID-19 status (Table 1).

In further analysis, the set of COVID-19 patients (n=472) was divided into survivors (n=350) and those who died of COVID-19 infection during hospitalization (n=122). Patients with COVID-19 who were discharged from hospital were significantly younger than non-survivors (66.0±15.7 vs. 76.3±10, p<0.001). The overall mortality rate of hospitalized patients with COVID-19 infection was 26 %, of which 55 % were women (n=67). COVID-19 positive patients who died (n=122) had significantly lower platelet number (p=0.006), lower plateletcrit (p=0.028) and on the statistical significance border, a greater degree of PLT anisocytosis expressed by the PDW parameter (p=0.057). However, the relationship demonstrating the highest statistical significance was between mortality in COVID-19 positive patients and the platelet size expressed by the MPV parameter and the P-LCR parameter (both p<0.001). Table 2 shows the relationship of selected platelet indices with mortality in COVID-19 positive patients.

The statistically significant relationship was between mortality in patients with COVID-19 (n=122) and MPV, P-LCR, PLT/MPV, PDW/PCT ratio and NLR (p<0.001, respectively), and PLT and PCT (p=0.006 and p=0.028, respectively).

A significant positive correlation was observed between mortality and MPV (OR 2.29; 95 % CI 1.70–3.08, p<0.001) and age (OR 1.06; 1.03–1.08, p<0.001) in multivariable logistic regression analysis (Table 3). When NLR was included into this model, MPV was stronger predictor of mortality (OR 2.48; 95 % CI 1.79–3.43, p<0.001) compared to NLR (OR 1.06; 95 % CI 1.03–1.08, p<0.001) and age (OR 1.04; 95 % CI 1.02–1.07, p<0.001) (Table 4).

## Discussion

Our study confirmed that COVID-19 positive patients had significantly larger platelets (expressed by higher MPV) and a higher percentage of platelets larger than 12 fl (expressed by higher P-LCR) than the patients without COVID-19. Numerous studies have demonstrated that markers such as MPV, and various platelet-related indices are altered in the context of systemic inflammation and are closely associated with disease severity across a broad spectrum of clinical conditions, including infections, cardiovascular diseases, autoimmune disorders, and malignancies [4–12].

Results of our research highlight MPV, a simple and routinely measured hematological parameter, as a strong and independent predictor of mortality in COVID-19. Its accessibility makes it a practical tool for real-world clinical use. There is evidence that increased MPV is associated with higher morbidity and all-cause mortality [5]. Elevated MPV reflects increased platelet activation and is considered a marker of systemic inflammation and disease severity [13].

Based on data from meta-analyses, we confirm the relationship between a lower platelet count and the severity of the COVID-19, which was expressed in mortality [6]. Deceased COVID-19 positive patients had significantly lower platelet count, lower plateletcrit, but higher MPV and P-LCR parameters. While both MPV and P-LCR reflect changes in platelet size, they represent different aspects of platelet morphology. MPV provides a continuous measure of the average platelet volume and is a well-established marker of platelet activation, inflammation, and disease severity. In contrast, P-LCR reflects the proportion of large platelets exceeding 12 fl, which may not capture the full variability of platelet size or subtle shifts in platelet activation.

In our study, the average MPV in deceased COVID-19 patients was 10.1±1.3 fl, substantially above the reported cut-off of 8.74 fl for severe inflammation [4]. This indicates that platelet enlargement was below the 12 fl threshold used in P-LCR calculation. Therefore, the lack of statistical significance for P-LCR was present in the multivariate logistic regression models for predictors of COVID-19 mortality, potentially overlooking relevant platelet size changes occurring below the 12 fl cutoff value.

Güçlü *et al.* reported COVID-19 mortality 8.4 times higher in patients who had oxygen saturation (pO_2_) under 90 % on admission and 1 unit increase in MPV increased mortality 1.76 times [14]. In our study 1 unit increase in MPV increased mortality 2.48 times in the multivariate regression model with NLR.

Our findings align with those of Mobarki *et al.*, who investigated the prognostic value of the MPV/PLT ratio and NLR in study sample of 110 COVID-19 patients. The deceased group of patients had a significantly elevated MPV/PLT and NLR as compared to the group of discharged patients [15].

While the sensitivities of MPV/PLT, PDW/PLT, MPV/PCT, and PDW/PCT indices were in Gozukucuk *et al.* study over 80 %, neutrophil and white blood cell sensitivities were found to be lower (74 % and 68.8 %, respectively) [16]. In Akhavizadegan *et al.* study, lymphocyte percentage, blood oxygen level, and platelet count were significantly higher in patients who had recovered from COVID-19 infection [17].

In the study of Yardimci *et al.*, mortality of patients with COVD-19 infection was associated with lower baseline lymphocyte counts, PLT, PCT, 3^rd^ day lymphocyte counts and PCT. Higher baseline CRP, LDH, ferritin, MPV/PCT, PDW/PLT, PDW/PCT and 3^rd^ day CRP, LDH, ferritin, procalcitonin, PDW, MPV/PCT, PDW/PLT, and PDW/PCT ratios were also associated with poor prognosis [18]. In our study dynamic changes in platelet indices over the course of hospitalization were not evaluated. This is possible limitation of the study as platelet indices were assessed at a single time point upon admission.

Our findings contrast with those of Polat and Demir, who reported no significant difference in MPV between outpatient and inpatient COVID-19 cases. This discrepancy may be attributed to differences in study design, patient populations, and outcome measures. While their study focused on admission-level triage decisions, our analysis evaluated mortality among hospitalized patients, where inflammatory and thrombotic processes may be more advanced. These differences highlight the importance of context when interpreting the prognostic value of platelet indices, particularly MPV [19].

The role of platelets in the severity of coronavirus disease requires further exploration. To determine whether the platelet indices are useful in predicting COVID-19 severity, additional scores were introduced (e.g., Fussa Platelet Score) and they need further evaluation [20].

The strength of the study is that it analyzes not just MPV, but also other platelet-related parameters (e.g., PLT, PCT, P-LCR, PDW/PCT, PLT/MPV), offering a broader view of platelet changes in COVID-19 and enhancing the depth of the analysis. The inclusion of a control group without systemic inflammation provides a clear contrast and increases the validity of the observed differences in platelet indices. By combining mean platelet volume (MPV) with the neutrophil-to-lymphocyte ratio (NLR) in the multivariate logistic regression, this study demonstrates that MPV has superior prognostic value for predicting mortality in hospitalized COVID-19 patients. This represents a novel and original contribution of our research.

Limitations of the study are single-center and retrospective design, which may introduce selection bias and limit the extent of the findings.

Incorporating platelet indices into clinical assessment may improve prognostic accuracy and guide treatment decisions in patients with COVID-19. MPV and related platelet indices, along with NLR, are not only valuable in COVID-19 but may serve as accessible, cost-effective markers for risk stratification in various acute and chronic inflammatory conditions (sepsis, pneumonia, acute pancreatitis, etc.). Their integration into clinical evaluation may help predict disease severity, guide treatment intensity, and improve clinical outcomes. Comparative studies on MPV across multiple inflammatory diseases are needed in future research.

## Conclusions

This study demonstrated that MPV had superior prognostic value to NLR and age in predicting poor outcomes, highlighting its potential as a simple, accessible biomarker for early risk stratification of hospitalized COVID-19 patients.

## Figures and Tables

**Table 1:** Relationship of selected platelet indices in patients with COVID-19 and non-COVID-19.

	COVID-19 (n=472)	non-COVID-19 (n=100)	p
*PLT (×10* *^9^* */l)*	224.7±105.9	240±62.6	0.156
*PCT (%)*	0.26±0.1	0.26±0.06	0.945
*MPV (fl)*	9.2±1.4	8.8±1.2	0.002
*PDW (%)*	16.9 (16.5;17.3)	16.9 (16.4;17.3)	0.428
*P-LCR (%)*	33.7±8.9	24.8±7.1	0.002

PLT – platelets, PCT – plateletcrit, MPV – mean platelet volume, PDW – platelet distribution width, P-LCR – platelet large cell ratio. Data are presented as mean ± SD, PDW as median, 25^th^, and 75^th^ percentile.

**Table 2:** Relationship of selected blood count parameters and platelet indices with mortality in patients with COVID-19 infection.

	Non-survivors (n=122)	Survivors (n=350)	p
*PLT (×10* *^9^* */l)*	201.2±111.1	232.6±103.1	0.006
*MPV (fl)*	10.1±1.3	8.9±1.3	<0.001
*PCT (%)*	0.24±0.09	0.26±0.10	0.028
*PDW (%)*	17.1 (15.7;17.6)	16.9 (16.5;17,3)	0.057
*P-LCR (%)*	36.5±8,2	32.8±9	<0.001
*MPV/PLT*	0.05 (0.03;0.09)	0.04 (0.03;0.06)	<0.001
*PDW/PCT*	0.05 (0.03;0.09)	0.04 (0.03;0.05)	<0.001
*NLR*	8.26 (4.93;21.63)	5.26 (3.10;9.25)	<0.001

PLT – platelets, PCT – plateletcrit, MPV – mean platelet volume, PDW – platelet distribution width, P-LCR – platelet large cell ratio, PLT/MPV – platelet to mean platelet volume ratio, PDW/PCT – platelet distribution width to plateletcrit ratio, NLR – neutrophil to lymphocyte ratio. Data are presented as mean ± SD, PDW and NLR as median, 25^th^ and 75^th^ percentile.

**Table 3:** Multivariable logistic regression analysis of mortality in patients with COVID-19.

	Mortality in COVID-19+	
	OR (95 % CI)	p
*Age (years)*	1.06 (1.03–1.08)	<0.001
*PLT (×10* *^9^* */l)*	1.0 (0.99–1.01)	0.325
*MPV (fl)*	2.29 (1.70–3.08)	<0.001
*PCT (%)*	0.004 (0.0–2.87)	0.101
*PDW (%)*	1.07(0.91–1.26)	0.395
*P-LCR (%)*	0.97 (0.92–1.02)	0.236

PLT – platelets, PCT – plateletcrit, MPV – mean platelet volume, PD – platelet distribution width, P-LCR platelet large cell ratio. Data are presented as odds ratio (OR), 95 % confidence interval (CI).

**Table 4:** Multivariable logistic regression analysis of mortality in COVID19+ patients. Model with neutrophil-to-lymphocyte ratio (NLR).

	Mortality in COVID-19 +	
	OR (95 % CI)	p
*Age (years)*	1.04 (1.02–1.07)	<0.001
*PLT (×10* *^9^* */l)*	1.0 (0.99–1.01)	0.223
*MPV (fl)*	2.48 (1.79–3.43)	<0.001
*PCT (%)*	0.002 (0.0–0.13)	0.023
*PDW (%)*	1.02 (0.86–1.21)	0.860
*P-LCR (%)*	0.97 (0.92–1.03)	0.302
*Ne/Ly ratio (NLR)*	1.06 (1.03–1.08)	<0.001

PLT – platelets, PCT plateletcrit, MPV – mean platelet volume, PDW – platelet distribution width, P-LCR – platelet large cell ratio, NLR – neutrophil to lymphocyte ratio. Data are presented as odds ratio (OR), 95 % confidence interval (CI).
